# Aβ plaques induce local pre-synaptic toxicity in human iPSC-derived neuron xenografts

**DOI:** 10.1016/j.stemcr.2025.102754

**Published:** 2026-01-02

**Authors:** Jacqueline Fréderique Maria van Vierbergen, Carles Calatayud, Sriram Balusu, Nicolò Carrano, Nicolas Peredo, Katlijn Vints, Sandra Fernández Gallego, Katrien Horré, Bart De Strooper, Patrik Verstreken

**Affiliations:** 1 VIB KU Leuven Center for Brain & Disease Research, Leuven, Belgium; 2 KU Leuven, Department of Neurosciences, Leuven Brain Institute, 3000 Leuven, Belgium; 3 VIB BioImaging Core, 3000 Leuven, Belgium; 4 UK Dementia Research Institute, University College London, London, UK

**Keywords:** xenotransplantation, pre-synapse, Alzheimer’s disease, synapse toxicity, extracellular Aβ, dystrophic neurite, amyloid-beta plaque

## Abstract

Xenotransplantation enables the interrogation of human neuron-specific vulnerabilities to Alzheimer’s pathology within a physiologically relevant *in vivo* context. While amyloid-beta (Aβ) is known to disrupt synaptic integrity, it remains uncertain whether the synaptotoxicity observed *in vitro* accurately models the disease. Here, we establish a xenotransplantation paradigm in which human neurons integrate into the brains of amyloid precursor protein (APP) transgenic mice that develop amyloid plaques. Using a genetically encoded pre-synaptic reporter, we label human pre-synapses post engraftment to assess early-stage pathology. We demonstrate that extracellular Aβ plaques induce localized synaptic damage in human neurons, characterized by local pre-synaptic loss and the formation of dystrophic neurites. Notably, this pathology is restricted to the plaque microenvironment and does not result in widespread pre-synaptic degeneration. Our findings establish this human-mouse chimera model as a platform for dissecting Aβ-induced synaptic pathology and reveal that extracellular Aβ exerts compartmentalized yet impactful toxicity on human pre-synapses.

## Highlights

• Human pre-synapses are genetically tagged and quantified after xenotransplantation
• Transplanted human neurons integrate and form synapses throughout the mouse brain
• Amyloid plaques induce dystrophic neurite formation in human neurons
• Extracellular Aβ plaques cause local loss of human pre-synaptic terminals

## Teaser

Verstreken and colleagues provide a modified human iPSC line that enabled the visualization of human pre-synaptic terminals post engraftment in the mouse brain. This allowed for the evaluation of early-stage deficits induced by extracellular Aβ pathology, specifically pre-synaptic loss. They found the occurrence of dystrophic neurites in response to amyloid and a local plaque-specific pre-synaptic toxicity without observing overt pre-synaptic loss.

## Introduction

Modeling brain disorders remains a significant challenge, as mouse models often fail to fully recapitulate human-specific disease pathology, particularly in complex neurodegenerative conditions such as Alzheimer’s disease (AD). Emerging evidence has highlighted the importance of species-specific factors in the pathogenesis of AD (Balusu et al., 2023; Espuny-Camacho et al., 2017; Kwak et al., 2020; Mancuso et al., 2024), indicating the necessity of incorporating a human genetic background in modeling such disorders. Over the last few years, substantial progress has been made in the development of human stem cell-based models, including brain organoids and three-dimensional cultures, which has advanced our understanding of human-specific vulnerabilities to diseases (Batenburg et al., 2023; Fernandes et al., 2024; Kwak et al., 2020). Despite this, modeling neurodegeneration *in vitro* remains challenging, particularly to recapitulate the cell diversity as well as the complex pathophysiology that occurs in the human brain. As a result, discrepancies persist between *in vivo* mouse models and *in vitro* human systems, causing divergent findings regarding the toxicity of aggregation-prone proteins implicated in neurodegenerative diseases (Bassil et al., 2021; Ruiter et al., 2021). Xenotransplantation models offer the use of human genetic architecture while residing in an *in vivo* environment (Levy and Paşca, 2025). However, model-specific limitations still restrict the use of investigating early-stage defects; for instance, it is difficult to evaluate in these models the effects on mouse versus human synapses.

It is currently still a topic of active debate how AD aggregation-prone proteins, i.e., amyloid-beta (Aβ) and Tau, contribute to cognitive decline (Gallego-Rudolf et al., 2024; Karran and De Strooper, 2016). In particular, the cellular mechanisms by which Aβ species exert toxicity on synapses remain unclear (Ferreira and Klein, 2011; Freir et al., 2011; Hong et al., 2016; Walsh and Selkoe, 2007; Zhang et al., 2022). In postmortem AD brain, Aβ42 was found to accumulate in both pre- and post-synaptic sites, leading to abnormal synapse morphology (Koffie et al., 2012; Gouras et al., 2005; Takahashi et al., 2002). Widely utilized AD models incorporate Familial Alzheimer's disease (FAD) mutations, which affect amyloid precursor protein (APP) processing and lead to both intracellular and extracellular accumulation of Aβ. The broad range of induced effects—such as changes in APP cleavage and the diverse Aβ aggregation patterns—makes it challenging to pinpoint the precise mechanisms by which Aβ exerts its synaptotoxic effects. Methods to study the specific effects of extracellular Aβ typically rely on the injection or addition of oligomeric Aβ in *in vitro* cultures or in mouse models. While such strategies induce synaptic and neuronal loss *in vitro* and alter electrophysiological responses *in vivo* (Bassil et al., 2021; Shankar et al., 2008), it remains uncertain whether these models faithfully replicate the human disease pathology.

Xenotransplantation models, in which human stem-cell-derived neurons are transplanted into mouse brains, offer a promising avenue for studying human-specific neuronal vulnerability within an *in vivo* environment. An AD xenotransplantation model has been developed in which wild-type human neurons are transplanted into amyloid-producing mice, enabling the investigation of early Aβ-induced toxicity in human neurons (Balusu et al., 2023; Espuny-Camacho et al., 2017). A current limitation of this model, however, is the inability to visualize and trace human pre-synapses following transplantation. To address this, we genetically modified the Kolf2.1J human induced pluripotent stem cell (iPSC) line to express HA-tagged Synaptophysin, allowing for the specific tracking of human pre-synapses *in vivo*. Human neural progenitors were engrafted into Aβ-producing mice, enabling the assessment of exogenous Aβ on human pre-synapses devoid of disease-causing mutations. This allows us to study the earliest stage deficits in a disease-relevant environment. We find that human axons become dystrophic upon contact with Aβ plaques, accumulating Synaptophysin, lysosomal marker LAMP1 (lysosomal-associated membrane protein 1), and markers of hyperphosphorylated Tau. Additionally, we observe a significant local loss of human pre-synapses near Aβ plaques, although overall synapse density across the human neurons remains unaffected. These findings demonstrate that extracellular Aβ induces localized, plaque-specific effects on human pre-synaptic terminals and provide a valuable model to study human pre-synaptic terminals *in vivo*, also in the context of disease.

## Results

### Human engineered cells to visualize pre-synaptic terminals

**Figure 1:**
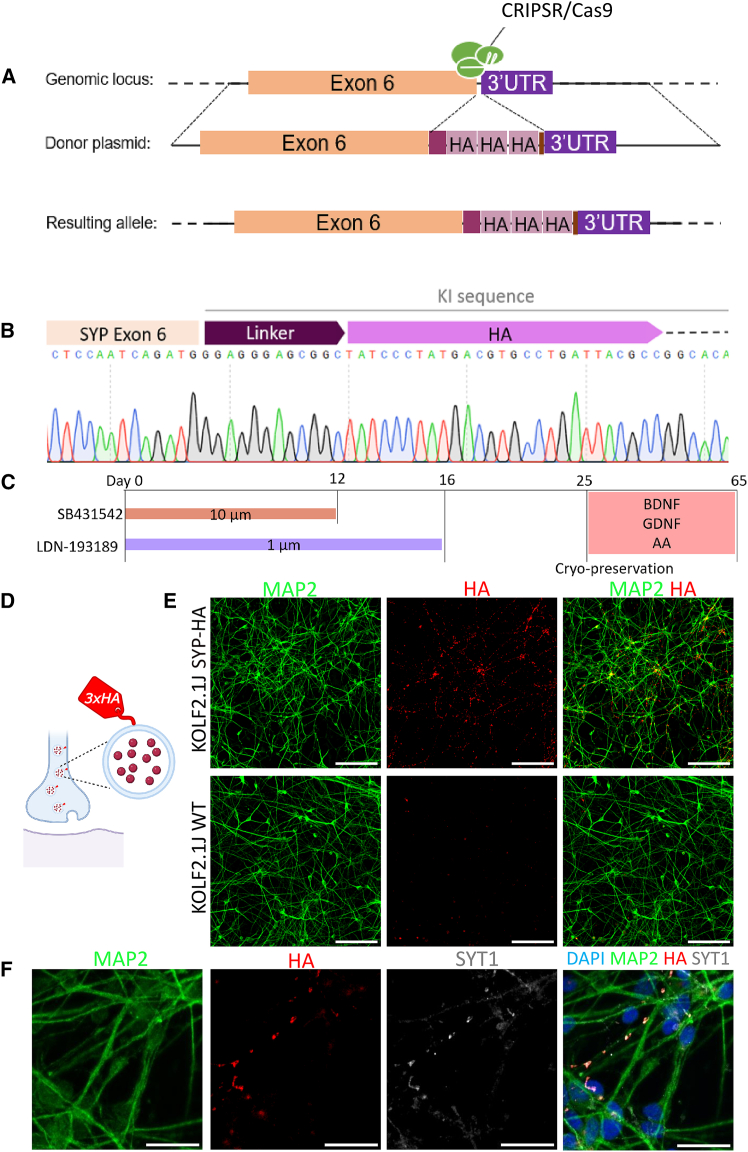
Labeling of endogenous *synaptophysin* with an HA tag using CRISPR-Cas9 (A) Schematic of CRISPR-Cas9 gene editing of endogenously tagged *SYP*-3×HA at exon 6. Donor plasmid contains a linker sequence followed by a triple HA-tag, the solid and dashed lines represent a *SYP* homology arm and genomic DNA, respectively. (B) Sanger sequencing of Kolf2.1J *SYP*-HA cells showing the 5′ junction and *SYP* exon 6 followed by the knockin sequence (linker and 3×HA tag). (C) Cortical NPC induction protocol using dual-SMAD; see also Figure S1. (D) Schematic of HA-tagged synaptic vesicles. (E) HA immunostaining indicating specificity to human neurons tagged with *SYP*-HA versus the unedited human neurons D50 (scale bars: 100 μm). (F) Kolf2.1J *SYP*-HA neurons D95 show colocalization of HA with the pre-synaptic marker Synaptotagmin-1 (scale bars: 20 μm).

There are to date no human pre-synapse-specific antibodies that we are aware of that do not cross-react with their murine equivalents. To study and quantify human pre-synapses after transplantation of human neurons in the mouse brain, we used CRISPR-Cas9 to genetically engineer the Kolf2.1J iPSC line and inserted 3 copies of the well-characterized hemagglutinin (HA) tag into the *synaptophysin* locus. This modification results in a C-terminally tagged pre-synaptic vesicle-associated protein (Chantranupong et al., 2020) (Figures 1A and 1D). Successful editing was confirmed using Sanger sequencing (Figure 1B) and comparative hybridization arrays to rule out medium- to large-sized chromosomal aberrations (Figure S1A).

To assess if the engineered cells express tagged Synaptophysin, we generated cortical neurons using the dual-SMAD inhibition protocol (Chambers et al., 2009) followed by DAPT treatment and conducted immunohistochemical analyses (Figures 1C and S1C). We observed strong and specific anti-HA labeling that colocalizes with anti-Synaptotagmin-1 labeling, a well-characterized pre-synaptic marker (Brunger et al., 2019) (Figures 1E and 1F).

### Human neurons integrate into the mouse brain and form mature synaptic contacts

**Figure 2:**
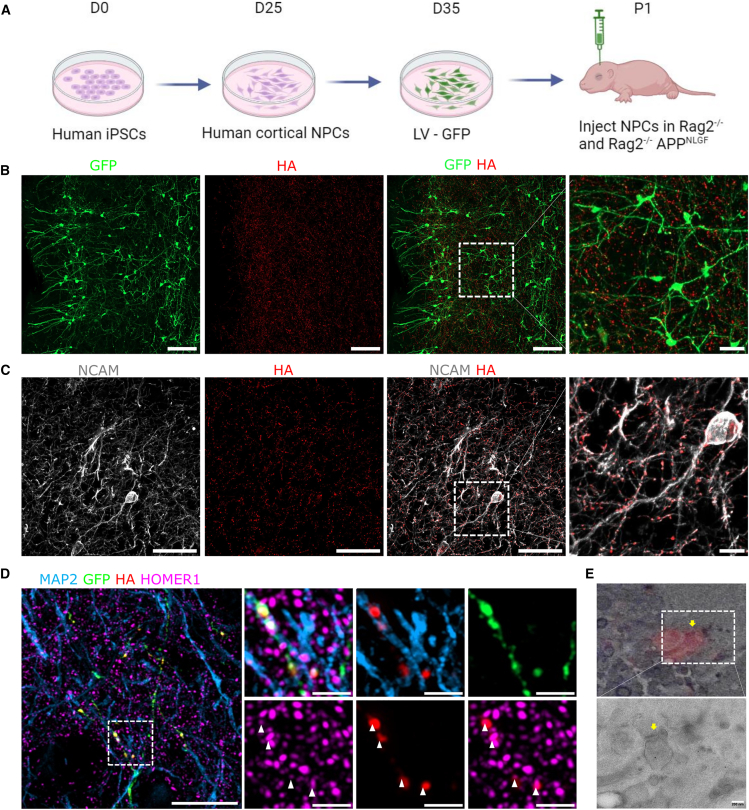
Human neurons integrate as single neurons into the mouse brain and form pre-synaptic contacts with mouse neurons (A) Schematic of xenotransplantation procedure. (B) Human neurons (GFP^+^) integrated in the mouse cortex and show a complex network of pre-synaptic puncta (SYP-HA) at 6 MPT in control mouse, analyzed for both control (*n* = 3) and amyloid (*n* = 3) mice (scale bars: 100 and 25 μm in the inset). (C) HA^+^ pre-synaptic puncta colocalize with human processes positive for the human-specific process marker (NCAM^+^) at 6 MPT in a control mouse, analyzed for both control (*n* = 3) and amyloid (*n* = 3) mice (scale bars: 50 and 10 μm in the inset). (D) Human axon (GFP^+^) forms contacts with a mouse dendrite (GFP- MAP2^+^) and makes synaptic contact (HA, Homer1 colocalization) at 12 MPT in control (*n* = 3) and amyloid (*n* = 3) mice; white arrowheads indicate synaptic contact sites between human and mouse neurons in the control mouse (scale bars: 5 μm and zoom: 2 μm). See also Figure S6. (E) Correlative light-electron microscope of Alexa nanogold labeling of HA at 6 MPT in control mice (*n* = 1); yellow arrowhead indicates the pre-synaptic site (scale bars: 200 μm).

To study human synapses exposed to exogenously produced Aβ, we xenotransplanted human neuronal progenitors (NPCs) into mice with a combination of AD pathogenic mutations in their *App* gene (*App*^*NL-G-F*^). The *App*^*NL-G-F*^ mouse model expresses the humanized Aβ under the endogenous *App* promoter of the mouse, resulting in plaque formation from 1 to 2 months of age. We first virally transduced the NPCs to express GFP enabling us to identify the transplanted neurons *in vivo*. We injected the cells bilaterally in close proximity to the cortex of immunodeficient control (*Rag2*^−/−^) or Aβ-producing mice (*Rag2*^−/−^, *App*^*NL-G-F*^) in P1/P2 pups (Figure 2A) (Balusu et al., 2023) and analyzed them 6 and 12 months post transplantation (MPT) using immunohistochemistry.

The transplanted Kolf2.1J SYP-HA NPCs differentiate predominantly into neurons (GFP^+^ NEUN^+^) and oligodendrocytes (GFP^+^ Olig2^+^). Both cell types integrate into the mouse brain (Figure S2A) and are also present in the core graft (Figure S2B). The core graft region (GFP^+^) is typically located below the cortex, in the septal nucleus of the stria terminalis (Figures S2C and S5). We also find, to a lesser extent, human astrocytes derived from the NPCs, detected by positive labeling with human-specific astrocyte marker (Stem123^+^) that co-stained with human nuclear marker (HUNU^+^) (Figures S2C and S2D).

Transplanted neurons matured, expressing both 3R and 4R Tau in the core grafts at 6 MPT and 12 MPT (Figure S3A), and indirectly, we detected neuronal activity through positive labeling of immediate-early gene c-Fos (Figure S3B). Approximately 80% differentiated into upper layer cortical neurons (SATB2^+^), which remain stable over time and after amyloid exposure (Figures S3C and S3D). Moreover, these neurons are predominantly excitatory, as indicated by colocalization of VGLUT1 rather than VGAT with the HA-labeled human pre-synaptic puncta (Figure S4).

These neurons can integrate in the mouse brain at sites away from the core-graft region (Figure 2B). We used anti-HA and anti-GFP labeling to reveal human pre-synapses and found a dense network of pre-synaptic sites throughout the host as well as specifically in the cortex (Figures 2B and S5). The analyzed neurons are human, as they are positive for the human-specific neuronal process marker NCAM/CD56. These neuronal processes are also abundantly decorated with HA-labeled pre-synaptic sites, indicating the specificity of our tool following transplantation (Figure 2C).

Next, we assessed whether human pre-synapses form post-synaptic contacts with mouse or other human neurons in the cortex. We employed Airyscan super-resolution microscopy and labeled xenotransplanted mouse brain slices with anti-HA, the post-synaptic marker Homer1 , and the dendritic neurite marker MAP2. This reveals numerous GFP- and HA-positive human axons that make contact with MAP2-positive GFP-negative dendrites (Figures 2D and S6). Moreover, human pre-synapses (HA^+^) are also found to colocalize with post-synaptic Homer1-labeled puncta, indicating mature and connected synapses.

We utilized correlative light and electron microscopy to examine the ultrastructure of synapses with human pre-synaptic compartments. Alexa nanogold-labeled HA puncta were identified, employing a resin-embedding protocol to preserve fluorescence for precise correlation in ultrathin sections (see methods). Sections were first imaged using confocal microscopy to confirm the targeted region and localize HA puncta, followed by imaging with transmission electron microscopy to align fluorescence with gold particles and confirm synaptic structures. Human pre-synaptic sites identified through this approach are observed in contact with distinct morphologically characterized post-synaptic partners (Figure 2E).

These findings show that human neurons mature well, integrate into the mouse brain, and form synaptic contacts with other neurons. This provides a physiologically relevant model to study how amyloid affects human pre-synapses in an *in vivo* environment.

### Human neurons grafted in the *App*^*NL-G-F*^ mouse show axonal dystrophies containing Synaptophysin, hyperphosphorylated Tau, and lysosomal proteins

**Figure 3:**
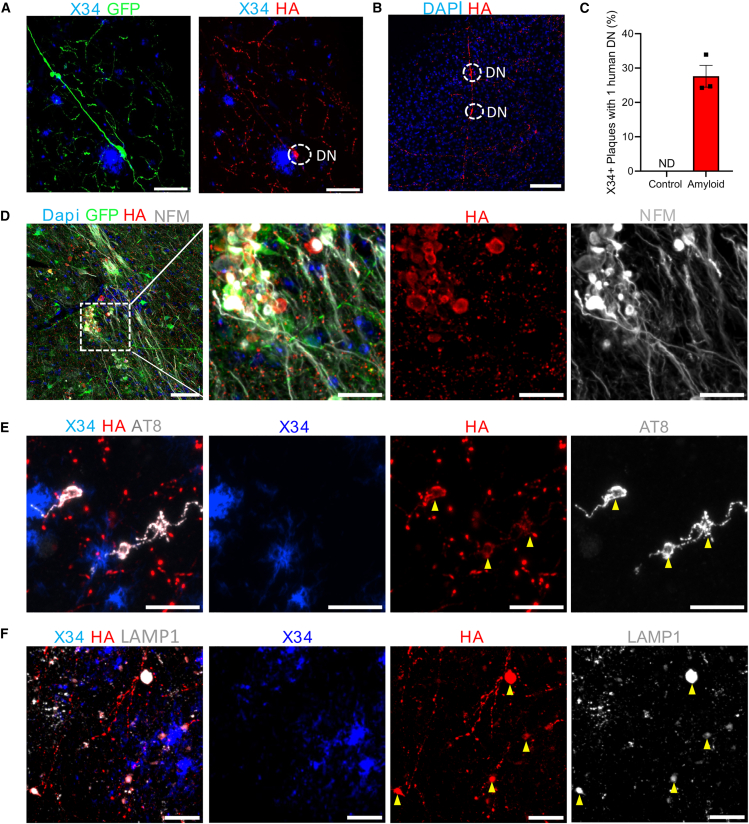
Human axons show dystrophies when in contact with dense-core plaques (A) Human neuron shows axonal swelling (GFP^+^ SYP-HA^+^) when in close contact with an Aβ plaque at 6 MPT in amyloid (*n* = 3) mice (scale bars: 25 μm). (B) Dystrophic axonal swellings (SYP-HA^+^) in human axon in amyloid (*n* = 4) mice at 12 MPT (scale bars: 100 μm). (C) Around 30% of plaques contain at least one dystrophic neurite in a human synaptic area in amyloid (*n* = 3) mice at 6 MPT. (D) Dystrophic neurites co-stain with the axonal marker neurofilament medium in amyloid mice at 6 MPT (*n* = 3) and 12 MPT (*n* = 4) (scale bars: 50 μm and inset: 20 μm). (E) Dystrophic neurites are positive for hyperphosphorylated Tau (AT8^+^) at 6 MPT (*n* = 3) and 12 MPT (*n* = 4); yellow arrowheads indicate that dystrophic neurites colocalize with hyperphosphorylated Tau (scale bars: 10 μm). (F) Human DNs are also positive for lysosomal marker LAMP1 stained with human-specific antibody at 6 MPT (*n* = 3) and 12 MPT (*n* = 4); yellow arrowheads indicate human dystrophic neurites (scale bars: 25 μm). See also Figure S6.

Human neurons transplanted in *App*^*NL-G-F*^ mice are exposed to Aβ pathology starting from 2 months. To ensure proper maturation as well as long-term exposure of amyloid to the human neurons, we investigated mice older than 6 months, up to 12 months (Balusu et al., 2023; Espuny-Camacho et al., 2017). We examined whether Aβ pathology had any effect on human axonal health and pre-synaptic morphology. We found human axonal swellings that label positive for anti-HA when in close contact with Aβ plaques, resembling dystrophic neurites (DN) (Figure 3A). Axons do not seem to immediately degenerate when they contain axonal dystrophies, as they have “normal” morphology at locations beyond the axonal swellings (Figure 3B). Around 30% of Aβ plaques in the cortex that reside in a human grafted region contain dystrophic neurites as defined by HA-labeled swellings (Figure 3C). To further confirm the dystrophic neurite pathology, we co-labeled our samples with markers typically found in AD patient brains. We observe that HA^+^ dystrophic neurites are also positive for neurofilament medium (Figure 3D), hyperphosphorylated Tau (AT8) (Figure 3E), and human-specific lysosomal marker LAMP1 (Figures 3F and S7). Hence, our xenotransplantation of SYP-HA-labeled human neurons in an *App*^*NL-G-F*^ background recapitulates aspects of AD patient pathology. Noteworthy, due to the low abundance of human neurons versus host, we observe single-neuron dystrophies that have a different morphology than the multi-neuron PANTHOS (poisonous flower)-like pattern commonly described in AD models and patients (Lee et al., 2022).

**Figure 4:**
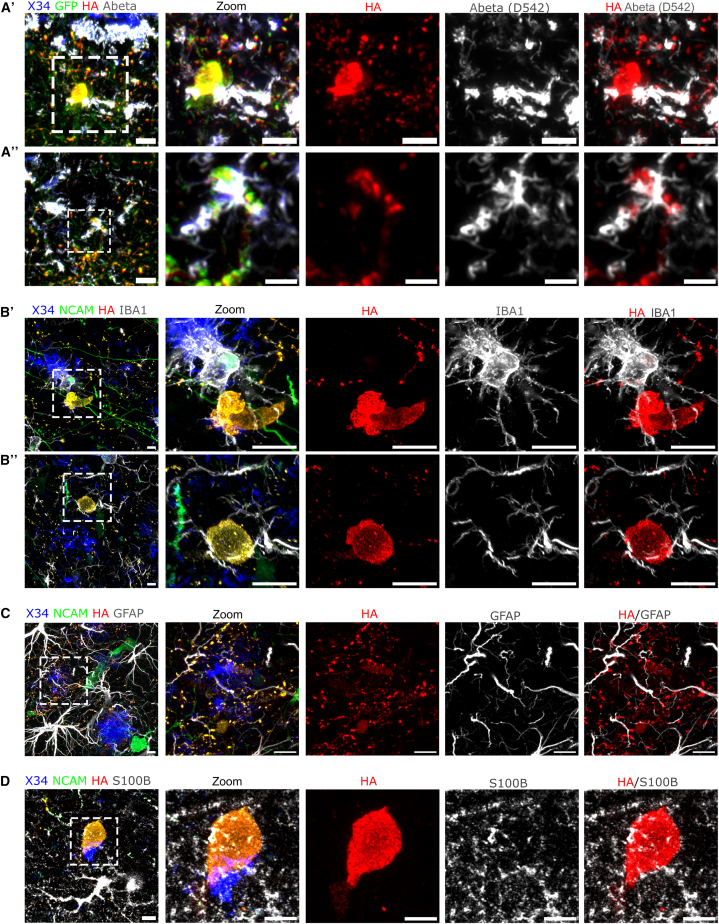
Aβ peptides and glia surround human dystrophic neurites (A and B) (A) Aβ peptides labeled with D542 are found in close contact to dystrophic neurites (HA^+^ swellings) (scale bars: 5 μm, A′: 5 μm and A″: 2.5 μm) at 12 MPT (*n* = 4 mice). (B) Microglial processes (IBA1^+^) are found to interact with human dystrophic neurites (HA^+^) (scale bars: 5 μm). (C and D) (C) Astrocytic processes (GFAP^+^) (D) and astrocytic cytoplasm (S100B^+^) labeling show close interaction with dystrophic neurites (HA^+^) (scale bars: 5 μm) at 12 MPT in amyloid mice (*n* = 4 mice).

To investigate in more detail the accumulation of Aβ pathology, we performed super-resolution microscopy. We find that human axons are surrounded by closely apposed “sticky” Aβ peptides (D54D2^+^) that appear most closely associated but not substantially inside the dystrophic neurites (Figures 4Aʹ and 4A″). Interestingly, we observed that microglial processes closely enwrap dystrophic neurites (Figures 4Bʹ and 4B″). We assessed whether the surrounding microglia that interact with DN exhibit a disease-associated microglia (DAM) signature by staining for DAM marker C-type lectin domain family 7 member A (Clec7a). We find that the microglia express Clec7a to varying extents, indicating a heterogeneous DAM-associated response (Figure S8). Next, we assessed whether dystrophic neurites and human pre-synapses are tagged for microglia engulfment. We labeled for complement component C1Q and found human pre-synaptic puncta colocalizing with C1Q, indicating that a subset of synapses are complement tagged for potential microglial recognition (Figure S9A, yellow arrows). Interestingly, we did not detect dystrophic neurites that are co-labeled with C1Q (Figure S9B), where the C1Q labeling is found to be predominantly located around the Aβ plaques (Figures S9B and S9C). Finally, astrocytic processes were found in contact with both human pre-synapses and dystrophic neurites (Figures 4C and 4D), indicating that glial cells are closely associated with neurite dystrophies.

### Plaque pathology induces local pre-synaptic loss in human neurons

**Figure 5:**
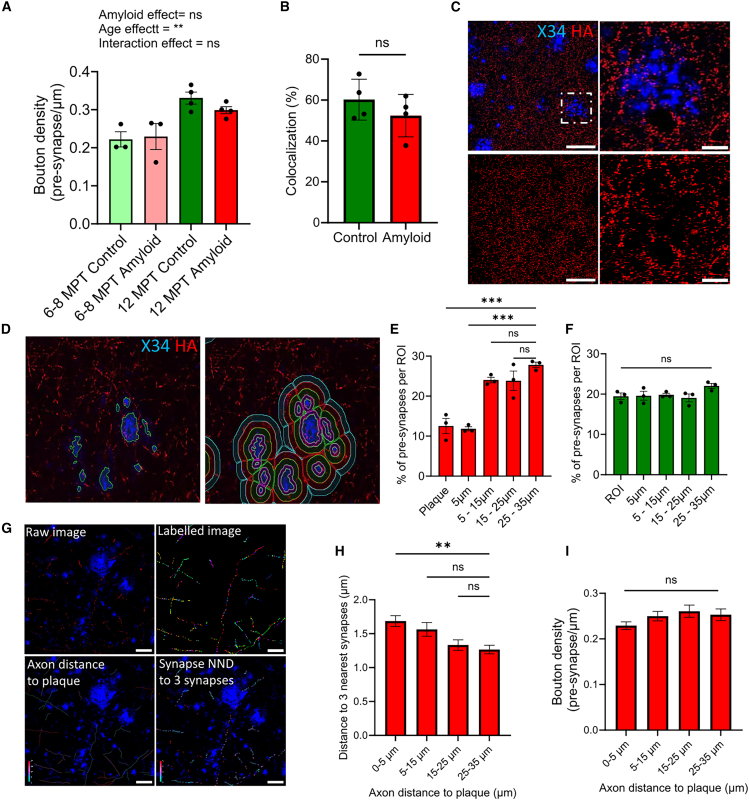
Aβ pathology affects pre-synaptic health locally but does not induce global changes in pre-synapse number (A) Bouton density of human axons in control and amyloid mice at 6–8 MPT (*n* = 3 and *n* = 3, respectively) and 12 MPT (*n* = 4 and *n* = 4, respectively). Statistical analysis was performed using two-way ANOVA. No significant main effect was detected for amyloid exposure comparing bouton density in control vs amyloid mice: F (1, 10) = 0.3840 P = 0.5493. A significant main effect was found for age comparing bouton density at 6-8MPT vs 12MPT: F (1, 10) = 20.22 P = 0.0011. No significant interaction effect was detected (Amyloid∗Age) F (1, 10) = 0.9992 P = 0.3411 (ns *p* > 0.5, ∗∗*p* < 0.01), sum of axonal length per mouse to caclulate bouton density > 1,000 μm. (B) HA^+^ puncta colocalized with Homer1^+^ puncta normalized to all HA^+^ puncta and displayed in percentages for control (*n* = 4) and amyloid (*n* = 4) mice at 12 MPT (>190 pre-synaptic puncta per mouse, unpaired *t* test F (3, 3) = 1.078 P = 0,9521, ns *p* > 0.5). (C) Aβ plaques stained with X34 surrounded by human pre-synapses (HA^+^) (scale bars: 50 μm and zoom is 25 μm). (D) Automated analysis using NIS software of synapse density surrounding dense-core plaques. (E) Pre-synaptic loss within the plaque and first 5 μm^2^ represented as percentage normalized to ROI area and total number of pre-synapses measured for each mouse (*n* = 3 mice and >50 plaques per mouse, age 6–8 MPT). Repeated measure one-way ANOVA F (4, 8) = 20.15, P = 0.0003 with Dunnett’s multiple comparisons test (25-35 µm vs. Plaque: P = 0.0005; 25-35 µm vs. 5 µm: P = 0.0004) (ns *p* > 0.5, ∗∗∗*p* < 0.001). (F) Synapse distribution in control mice (*n* = 3 mice and >100 ROIs per mouse, age 6–8 MPT. Repeated measure one-way ANOVA F (4,8) = 1.620. P = 0.2599 (ns *p* > 0.5) (G) Example of synapse nearest neighbour distance (NND) to plaque analysis indicating the raw image, the labeled image containing the axons and pre-synapses and color-coded the distance of the synapse to the plaque and its nearest neighboring 3 synapses. (H) Axons passing within 5 µm of a plaque show an increased distance to their three nearest neighboring synapses. One-way ANOVA: F (3, 357) = 4.826, P = 0.0026; Dunnett’s post hoc test (25–35 µm vs. 0-5 µm: P = 0.0036). (ns p > 0.5, ∗∗ p < 0.001). Note that quantification requires axons to have a minimum of four synapses. (I) Overall pre-synaptic density is not altered in axons passing in close proximity to a plaque at 6–8 MPT. One-way ANOVA: F(3, 411) = 1.763, P = 0.1535; ns p > 0.5. Data represent individual axons (3 mice; >70 axons per mouse) (mean ± SEM).

Human neurons integrate into the mouse brain, form mature synapses, and are responsive to Aβ plaque pathology. We therefore assessed the potential toxicity of Aβ plaques on human synapses *in vivo.* We first determined whether extracellular Aβ pathology influences global pre-synaptic health. Therefore, we measured total pre-synaptic density, independent of plaque location, and we compared this to human neurons that were transplanted in control mice. We noted that GFP expression faded in some neurons over time. Therefore, for further quantification, we co-labeled all the samples with human-specific process marker (hNCAM) and used this to manually annotate our axons. Then, we measured pre-synaptic density using automated pre-synapse detection (see methods and Figure S10A). We did not detect a general pre-synaptic loss induced by extracellular Aβ pathology (Figure 5A). Next, we measured whether colocalization is affected by amyloid at 12 MPT. We quantified the proportion of HA^+^ Homer1^+^ colocalized puncta relative to the total number of HA-labeled pre-synaptic puncta (Figure S10B). We found no significant reduction in colocalization after exposure to amyloid (Figure 5B). Note that albeit small, a trend toward reduced levels of pre-synaptic puncta and colocalized puncta was detected at 12 MPT in human neurons transplanted into amyloid mice compared with controls.

Next, we assessed synaptic integrity around plaques and quantified HA-labeled synaptic density in concentric regions around X34-labeled plaques. Our analysis showed a clear loss of human pre-synapses within Aβ plaques and within the first 5 μm surrounding the plaque (amyloid *n* = 3) (Figure 5E). Conversely, areas without plaques showed an even distribution of pre-synapses, and we did not detect a sudden loss of pre-synapses in one particular location (control *n* = 3) (Figure 5F).

Subsequently, we investigated whether plaque-induced synapse loss translates into an overall reduction in synapse density on axons that pass in close proximity to Aβ plaques. To address this, we examined the distribution and density of pre-synapses of axons passing at varying distances from Aβ plaques (Figures 5C and 5D). Synapse distribution was assessed by measuring the distance from each bouton to its three nearest neighboring boutons along the same axon. (Figure 5G). Our findings reveal a significantly increased distance between a bouton and its three nearest boutons in axons passing close to the Aβ plaque (<5 μm) (*n* = 600 axons from 3 mice) (Figure 5H). However, this increase in inter-synaptic distance is modest, as it does not result in statistically significant reduction in the overall pre-synaptic density of axons passing near plaques (Figure 5I). These results indicate a regional-restricted toxic effect of extracellular Aβ plaques on human pre-synapses.

## Discussion

This study establishes a human iPSC-derived neuronal system engineered to enable selective labeling of pre-synaptic terminals following xenotransplantation, providing a powerful platform to study human-specific synaptic vulnerability *in vivo*. Using this model, we demonstrate that human neurons integrate into the mouse brain and form synaptic connections throughout the host circuitry. Importantly, we show that extracellular Aβ plaques, generated by the mouse brain, trigger a localized loss of human pre-synaptic terminals within 5 μm of the plaques and promote the formation of dystrophic neurites, enriched in synaptic and pathological markers without causing global pre-synaptic degeneration.

This paper sheds light on the ongoing debate whether the Aβ toxicity observed *in vitro* represents relevant human pathology or rather that it models the toxicity secondary to the specific (lack of) cellular and tissue environment. In those models, pre-synaptic loss is observed ranging from 15% up to 70% depending on Aβ load in both primary rodent and human neuronal cultures (Bassil et al., 2021; Izzo et al., 2014; West et al., 2015). Our findings suggest that the strong synapse loss observed in these models may be due to an elevated Aβ load or the absence of an *in vivo* environment, which highlights the need for optimized AD models that recapitulate patient-specific and *in vivo* features.

Our data indicate that extracellular Aβ pathology does not lead to widespread pre-synaptic loss in this model. This is consistent with the mild changes induced by Aβ pathology on mouse inhibitory boutons (Ruiter et al., 2021). Similarly, in postmortem brain tissue from Alzheimer’s patients, plaques also coincided with local synapse loss; however, the radius around the plaque where synapse loss occurred was larger than in our xenotransplantation model (Koffie et al., 2012). This discrepancy is likely due to the advanced stage of the disease in postmortem analyses or to the older age of the neurons, which could lead to increased sensitivity to the plaque environment. Therefore, it would be interesting to boost the maturation of the human neurons and investigate whether this affects synapse vulnerability to amyloid pathology.

This work reveals the toxic effect of Aβ plaques on synapse integrity in relatively young human neurons; indeed, transplanted human neurons mature following their autonomously defined pace instead of following the (faster) mouse neuron maturation timeline (Linaro et al., 2019). Our work also shows that exogenously produced Aβ is sufficient to cause local synapse loss in human neurons. Moreover, dystrophic neurites are caused by Aβ plaques. It is unknown if dystrophic neurites are caused directly by Aβ or indirectly through microglia (Baligács et al., 2024; Yuan et al., 2022). Microglia depletion in an amyloid mouse model at an early age results in both decreased Aβ plaque size and dystrophic neurite formation whereas depletion at a later stage leads to increased plaque area and dystrophic neurites size (Baligács et al., 2024), hinting toward the idea that dystrophic neurite formation is more strongly correlated to Aβ plaque size than induced by microglia; however, more work is needed to confirm this hypothesis.

Multiple studies have aimed to explain how Aβ affects neuronal health. For instance, Aβ peptides can affect neuronal signaling by directly interacting with NMDA receptors (Ortiz-Sanz et al., 2022; Taniguchi et al., 2022); however, this is a post-synaptic effect and does not easily explain the dystrophic neurite formation, which is found to be primarily axonal/pre-synaptic. In addition, Aβ plaques are thought to destabilize microtubules (Sadleir et al., 2016). This defect will lead to impaired organellar transport, resulting in the accumulation of lysosomes as well as synaptic vesicles, which we also observed in the human neurons. Next to transport impairments, previous research indicates that Aβ may induce a change in axonal conductance. It was found that Aβ can bind to pre-synaptic nicotinic acetylcholine receptors, causing increased pre-synaptic calcium levels in isolated hippocampal synaptosomes (Dougherty et al., 2003). Finally, some studies suggest that Aβ peptides could affect axon conductance through ion channel pore formation (Shirwany et al., 2007) or through interactions with the lipid bilayer (Sokolov et al., 2006). While the exact mechanisms of how Aβ affects axonal conductance requires further study, Aβ does induce hyperactivity in axons containing neurite dystrophies (Yuan et al., 2022). Our work assesses Aβ toxicity on pre-synaptic terminals in a quantitative manner; however, to determine whether extracellularly produced Aβ peptides lead to functional impairments in human neurons, it would be necessary to perform additional experiments, such as two-photon calcium imaging of synaptic sites *in vivo* (Queiroz Zetune Villa Real et al., 2023; Tu et al., 2014; Zhang et al., 2022). Finally, other forms of Aβ-induced neurotoxicity might be caused by cell-autonomous effects (Gouras et al., 2005; Ripoli et al., 2014; Takahashi et al., 2002). This could potentially be tested by introducing *PSEN1* mutations in human neurons transplanted into wild-type mice and assessing pre-synaptic integrity. This approach allows for the assessment of APP processing alteration on cell-autonomous mechanisms of neuronal health. The alternative hypothesis is that amyloid plaques induce neuroinflammation and the toxic effect on human neurons is caused by microglia rather than by a direct effect of Aβ (Leng and Edison, 2020; Lv et al., 2024).

The application of a xenotransplantation model allows for long-term monitoring of human neurons while they reside in an environment that provides nutrients at a physiological level as well as vascularization (Levy and Paşca, 2025). Despite strong advantages, there are limitations of the model as well as issues of variability. Graft location, size, and integration can vary between mice as well as between NPC batches. Emphasis should be on strict quality control during the preparation of the NPCs as well as post transplantation (1–2 months after grafting) (Paşca et al., 2024). Moreover, currently we are analyzing the effect of amyloid on the human neurons 6–12 months post transplantation, which is time-costly. Enhancing maturation to shorten the post-transplantation period would be both time- as well as cost-effective.

This study employs the KOLF2.1J cell line; a recent report indicates that this iPSC line has one functional allele for *JARID2* and *ASTN2*, genes that have previously been associated with neurodevelopmental disorders (Gracia-Diaz et al., 2023). We and others have not detected any aberrations in the development of the human neurons derived from this cell line and therefore do not expect this to cause confounding effects (Ryan et al., 2024).

Future studies should aim to resolve two key open questions in the field. First, the contribution of intracellular APP processing to neuronal vulnerability could be addressed by xenografting iPSC-derived human neurons carrying FAD mutations into wild-type host brains, thereby isolating cell-intrinsic pathogenic mechanisms. Second, functional consequences of Aβ exposure should be examined using two-photon calcium imaging to assess synaptic activity in human neurons exposed to intracellular versus extracellular Aβ *in vivo*.

More broadly, the use of human iPSC-derived neurons in xenotransplantation models represents a transformative approach for studying human-specific aspects of neurodegeneration. Unlike rodent models, which often fail to fully recapitulate the selective vulnerability and cellular context of human neurons, iPSC-based systems offer access to genetically defined, patient-relevant cells that can be studied within the complex architecture of the living brain. As these models continue to evolve, they hold significant promise for uncovering early pathophysiological events, identifying therapeutic targets, and bridging the translational gap between experimental systems and human disease.

## Methods

### Plasmids

The plasmid for homology directed repair of HA-tagged *synaptophysin* was prepared in the following way. The 5′ homology arm of *SYP* was amplified from genomic DNA extracted from the H9 embryonic stem cell line. A G-block containing the 3× HA sequence was ordered from Integrated DNA Technologies (IDT) (see supplemental methods). The 5′ homology arm and the 3× HA G-block were assembled into the pUC19 vector. Prior to assembly, the vector was linearized by digestion with AatII and NcoI restriction enzymes. Gibson assembly was performed using the NEBuilder HiFi DNA Assembly Master Mix (New England Biolabs). The assembled plasmid was transformed into *Escherichia coli* competent cells and incubated at 30°C for 24 h. Colonies were picked and sent for Sanger Sequencing.

### CRISPR-Cas9 genome editing

We performed CRISPR-mediated editing of the *synaptophysin* locus in human iPSCs. We followed the protocol previously described in Skarnes et al. (2019). Cells were nucleofected with the Cas9 sgRNA TTCTCCAATCAGATGTAGTC (Synthego). 800,000 cells were nucleofected with 10 μg Cas9 nuclease (Alt-R S.p. HiFi Cas9 V3, IDT), 8 μg sgRNA, and 15 μg plasmid, using the Lonza 4D-Nucleofector System (program CD118). After nucleofection, cells were plated in a 24-well plate. After around 1 week, iPSC colonies were manually picked and plated into a 96-well plate. Crude cell lysates were screened by PCR to amplify a genomic region containing the CRISPR target site, followed by Sanger sequencing of purified PCR products. Comparative genomic hybridization array was performed on genomic DNA isolated from clones with correct editing to check for chromosomal aberrations. The top 5 predicted off-target sites using CRISPOR have been sequenced, and no off-target mutations have been detected. Correct clones were stained for pluripotency makers.

### Mice

All mice experiments were approved by the ethical committee for animal experimentation of KU Leuven and executed in compliance with the ethical regulation of animal research*. App*^*NL-G-F*^ mice (Apptm3.1Tcs) were crossed with immunodeficient *Rag2*^−/−^ mice (*Rag2tm1.1cgn;* Jackson Laboratory, strain 008309) to generate *App*^*NL-G-F*^ *^∗^ Rag2*^*−/−*^ mice. *Rag2*^−/−^ mice with the mouse APP allele were used as control animals. Mice were housed in a specific opportunistic pathogen-free animal facility. Both sexes were used for all the experiments. Sex was evenly distributed between the groups; both control and amyloid included 1 female and 2 males at 6–8 MPT (*n* = 3 in total), and at 12 MPT, both groups comprised 3 females and 1 male (*n* = 4 in total). Mice were housed in groups (2–4 mice per cage) with *ad libitum* food and water under a 14-h light/10 h dark cycle at 21°C.

### Pluripotent stem cell culture and neuronal differentiation

Kolf2.1J human iPSCs with HA-tagged *synaptophysin* were used for all experiments. It should be noted that Kolf2.1J iPSCs have likely one functional allele of *JARID2* and *ASTN2*. Cells were maintained in Gibco StemFlex Medium on Geltrex-coated plates and passaged at 60%–80% confluence. Following thawing, iPSCs were expanded and used for neuronal differentiation after minimally two passages. On day −1, iPSCs were dissociated with accutase and plated at a concentration of 400,000 cells/cm^2^ on Matrigel-coated wells of 6-well plates in StemFlex medium supplemented with 10 μM Rho kinase inhibitor (RI). On day 0, media was changed to neuronal maintenance medium (NMM) supplemented with SB431542 (10 μM) and LDN-193189 (1 μM). NMM contained (Gibco Neurobasal-A Medium #10888022, DMEM/F12 with GlutaMAX #31331-093, Gibco B-27 Supplement [50×], minus vitamin A #12587010, Gibco *N*-2 Supplement [μX] #17502048, Thermo Scientific GlutaMAX Supplement #35050038, Gibco Penicillin-Streptomycin [10,000 U/mL] #15140148, insulin solution human Sigma-Aldrich #I9278, Gibco 2-mercaptoethanol [50 mM] #31350010, Gibco MEM non-essential amino acids solution [100×] #11140050, Gibco sodium pyruvate [100 mM] #11360070). Media was refreshed every day (3–4 mL per well). NMM contained SB431542 until day 12 and LDN-193189 until day 16. At day 25, neural progenitor cells were detached using Accutase, counted, and cryopreserved in NMM media containing 10% DMSO. NPCs were stained for NPC markers to confirm successful differentiation.

### Neural progenitor cell preparation for grafting

Nine days prior to grafting, NPCs were thawed and plated on a Matrigel-coated 6-well plate in NMM supplemented with RI, at a density of four million NPCs per well, and let to recover for 2 days. NPCs were infected with lentiviral particles encoding CAG-GFP at MOI 4. Puromycin selection was initiated 2 days post infection and spanned over 3 days with increasing concentrations: 0.25 μg/mL on the first day, followed by 0.5 μg/mL for the second and third day. Cells were let to recover for 1 day and then prepared for grafting. On the day of grafting, RevitaCell Supplement (Gibco; #A2644501) was added 1 h before detachment. Cells were detached using Accutase and resuspended in Leibovitz’s L-15 Medium (Thermo Fisher Scientific; #11415064) supplemented with glucose solution (34 mM) and RevitaCell. Viability was measured using trypan blue staining, and cells were counted and diluted to a concentration of 50,000 cells/μL. Cells were transferred in an Eppendorf on ice to the SPF facility for grafting.

### Grafting

Mating of mice was performed in a time-controlled manner. Pregnant females were housed two per cage with enough nesting material. Pups were grafted at P1-P2. For grafting, pups were briefly cryo-anesthetized, after which a small incision was made at the injection side, i.e., from bregma, −1 mm posterior and ±1 mm lateral. Using a 26G Hamilton syringe, 1 μL of solution was manually injected bilaterally near the cortex of the pups. In total, 100,000 neural progenitor cells were grafted in each pup (50,000 at each side). After the injection, pups were allowed to recover under a heat lamp at 37°C. Pups were weaned at 3 weeks of age, housed in cages with 2–4 mice, and sacrificed at 6–12 months of age.

### Brain sample processing and confocal image acquisition

Mice received an overdose of pentobarbital and were perfused with PBS, followed by freshly prepared 4% paraformaldehyde and 8% sucrose solution in PBS. Brains were stored in this PFA solution overnight at 4°C. The next day the brains were washed three times in PBS. Using a vibratome (Leica VT1000S), the brains were sliced in 40-μm-thick coronal free floating sections and stored for up to 2 weeks in PBS in 24-well plates or frozen in cryo-protectant solution (30% ethylene glycol, 30% glycerol, and 40% PBS) for long-term storage at −20°C. Immunostaining was performed (see supplemental methods).

Image acquisition was performed using either a Nikon NiE upright A1R microscope or a Nikon TiE inverted A1R microscope, both equipped with an HD resonant scanner. Images were obtained using a Nikon Plan Apo 20×/0.75 DIC N2 air lens, Olympus PlanApo *N* 60× Oil Microscope Objective (Nikon TiE A1R) and CFI Plan Apochromat VC 60XC WI (1.2 NA) (Nikon NiE A1R). Super resolution microscopy was performed using ZEISS LSM900 in Airyscan mode with a 63× Plan Apochromat (1.40) oil objective. Images were processed in the Fiji/ImageJ software (version 1.54f with Java 1.8.0_322 [64-bit]), Imaris (10.2), and NIS Elements Analysis software (5.46.06).

### Statistics

Results are displayed as mean ± standard error of the mean (SEM). One-way analysis of variance (ANOVA) was used when comparing multiple groups, with Dunnett’s multiple comparisons post hoc test when comparing all groups to one pre-determined group (Figures 5G and 5H) or with Sidak multiple comparisons test when performing only pre-determined comparisons (Figure 5A). Unpaired *t* test was used to assess synapse colocalization (Figure 5B). Repeated measures ANOVA was used for plaque-induced synapse loss (Figures 5E and 5F). GraphPad Prism was used for statistical analysis.

## Resource availability

### Lead contact

Requests for further information and resources should be directed to and will be fulfilled by the lead contact, Patrik Verstreken (patrik.verstreken@kuleuven.be).

### Materials availability

Produced materials are available upon request.

### Data and code availability

Code can be accessed via https://github.com/vib-bic-projects/202409_Synapse_Neurite_Quantificator/tree/main.

## Acknowledgments

We thank Amber Claes and Véronique Hendricks for breeding and taking care of the mice. We thank Nikky Corthout, Axelle Kerstens, Abril Escamilla Ayala, and Pablo Hernández Varas of the 10.13039/501100004727VIB BioImaging Core for their assistance and support in imaging. We thank the members of the Verstreken and de Strooper lab for input and discussion. Schematic illustrations (Figures 1C and 2A) were created with https://BioRender.com. This work was funded by the 10.13039/501100000781European Research Council grants (P.V. and B.D.S.), Methusalem grant from 10.13039/501100004040KU Leuven and the 10.13039/501100011878Flemish Government (P.V. and B.D.S.), Alzheimer Research Foundation (SAO-FRA) (P.V. and B.D.S.), the Fonds voor Wetenschappelijk Onderzoek 10.13039/501100011878Vlaanderen (P.V. and B.D.S.), 10.13039/501100004727VIB, 10.13039/501100004040KU Leuven, 10.13039/100000957Alzheimer’s Association and 10.13039/100016948Tau Consortium (P.V.), the 10.13039/100016608Rainwater Charitable Foundation (P.V.), 10.13039/100007625Cure Alzheimer’s Fund (P.V.), Alzheimer Research Foundation (P.V.), Koning Boudewijn Stichting (P.V.), Medical Research Grant (B.D.S.), 10.13039/501100022601the Queen Elisabeth Medical Foundation for Neurosciences (B.D.S.), the Opening the Future campaign of the Leuven Universitair Fonds (B.D.S.), and the 10.13039/100000957Alzheimer’s Association USA (B.D.S.). B.D.S. holds the Bax-Vanluffelen Chair for Alzheimer’s disease. J.F.M.v.V. is supported by a fellowship from the 10.13039/501100003130FWO.

## Author contributions

Conceptualization, J.F.M.v.V., B.D.S., and P.V.; methodology, J.F.M.v.V., C.C., S.B., N.C., S.F.G., N.P., K.V., K.H., B.D.S., and P.V.; investigation, J.F.M.v.V., C.C., S.B., N.C., S.F.G., N.P., K.V., K.H., B.D.S., and P.V.; writing, J.F.M.v.V., B.D.S., and P.V.; funding acquisition, J.F.M.v.V., B.D.S., and P.V.; supervision, B.D.S. and P.V.; all co-authors read and edited the manuscript.

## Declaration of interests

P.V. is the scientific founder of Jay Therapeutics. B.D.S. has been a consultant for Eli Lilly, Biogen, Janssen Pharmaceutica, Eisai, AbbVie, and other companies and is now consultant to Muna Therapeutics. B.D.S. is a scientific founder of Augustine Therapeutics and a scientific founder and stockholder of Muna Therapeutics.

## Declaration of generative AI and AI-assisted technologies in the writing process

During the preparation of this work, the authors used ChatGPT (OpenAI) in order to assist with grammar checking, text editing, and formatting during the preparation of this manuscript. After using this service, the authors reviewed and edited the content as needed and take full responsibility for the content of the publication.

## Supplemental information

Document S1. Figures S1–S10 and supplemental methods

Document S2. Article plus supplemental information
