# Supplementary material for: Aβ plaques induce local pre-synaptic toxicity in human iPSC-derived neuron xenografts
Source: Stem Cell Reports. 2026 Jan 2;21(1):102754. doi: 10.1016/j.stemcr.2025.102754 (PMC12925968; doi:10.1016/j.stemcr.2025.102754)
Supplement: Document S1. Figures S1–S10 and supplemental methods [file mmc1.pdf]

Stem Cell Reports, Volume *21*

Supplemental Information

Aβ plaques induce local pre-synaptic toxicity in human iPSC-derived neuron xenografts

Jacqueline Fréderique Maria van Vierbergen, Carles Calatayud, Sriram Balusu, Nicolò Carrano, Nicolas Peredo, Katlijn Vints, Sandra Fernández Gallego, Katrien Horré, Bart De Strooper, and Patrik Verstreken

Supplemental methods

Plasmids

PCR amplification was performed using the following primers: Forward primer (Syp_5HA_Fwd): CCGAAAAGTGCCACCTGACGTCCCGCATGCTCATTCCTGC reverse primer (Syp_5HA_Rv): atagggatagccgctccctccCATCTGATTGGAGAAGGAGGTGGGT.

The sequence of the G-block is as follows:

ggagggagcggctatccctatgacgtgcctgattacgccggcacaggatcctacccctatgatgtgcctgactacgctggcagcgccggataccct
tatgatgtgcctgattatgctTAGTCTGGTGAGTGACCGGCGAGCGGTGCGGCCAAGGAGGGTACAAG
GAAGGAGACGAGCGGGTCAGTGAACCAATAAGAGTCAGGGGTAGAGAGTACGTAAGGCG
TTTACTGGGGCAAGTAAGGACTGAAGTTTGAAGGAGCCAATCAAGAGGCAGAACTACCTG
CTAAGAGCTGAAAAACTAGCTAATGAATGGAGAAAAGAAGAATGGTGCTTCAAGATGGAC
AGAATGAAGAGCCAATGGGAGGGAGGTAGAAGGGCAACTCGGCGGAAAGGCGGGGGA
GAGAAGCAGAGCAACCAATAAAGGGAGGGAATATCGGGGGACTTGCGCAGTGGCGAATC
AGCGGAGCGCAAGAGCCACGGAAGCGACGAGGAGGTGGGTCTTAGAAGTGACTGATAAG
CGGAGCAAAAGGCCAGCAAAAG.

Immunostaining

For immunofluorescence, brain sections were blocked in PBS containing 5% Normal Goat Serum, 2% Bovin Serum Albumin and 0.20% Triton X-100 for one hour at room temperature on an orbital shaker. Brain sections that required X34 (Sigma #SML1954) staining, were permeabilized prior to blocking and then incubated with X34 solution for 20 minutes. The following primary antibodies Homer1 (SYSY #160003), Synaptotagmin-1 (1:SYSY #105011), Map2 (SYSY #188004), GFP (Abcam #ab13970), NCAM (Santa Cruz #sc-106), NFM (Abcam #ab254348), AT8 (Thermo Fisher Scientific #MN1020), LAMP1 (Abcam #ab278043), LAMP1 (Santa Cruz #sc-19992), HA 3F10 (Roche #11867423001), HA C29F4 (Cell Signaling Technologies #3724), OC (MilliporeSigma #AB2286), β-Amyloid (D54D2) (Cell Signaling Technologies #8243), VGLUT1 (Addgene #180087), CD68 FA-11 (Biorad #MCA1957T), VGAT (Santacruz #sc-365180), Clec7a R1-8G7 (Invivogen # mabg-mdect-2), C1Q (Abcam #ab182451), were diluted in blocking solution and added to the sections overnight at 4°C. The next day, the brain sections were washed in PBS + 0.2% Triton X-100 (PBST) at RT. Then the corresponding Alexa-conjugated secondary antibodies (1:500) were added for two hours at room temperature. Brain slices were mounted on glass microscope slides with Mowiol (Sigma-Aldrich) and dried at room temperature for 24 hours. After which, the slides were stored at 4°C prior to imaging.

Correlative light and electron microscopy

Mice received an overdose of Pentobarbital and were perfused with 4% PFA (EMS; #15714) and 0.1% Glutaraldehyde (EMS; #16220) in 0.1M PB and kept in fixative overnight at 4°C. After rinsing 3x with cold 0.1M PB buffer, 80µm vibratome sections were cut. Sections were incubated in 0.5% sodium borohydride (Sigma-Aldrich; #71320) for 30 minutes on ice and rinsed three times by 0.1M PB. Next, sections were blocked with a blocking buffer (1% BSA, 0.01% glycine (Sigma-Aldrich; G7126), 0.01% lysin (Sigma-Aldrich; L5501), 0.05% Triton X-100 (EMS; #22146), 0.1% cold water fish gelatin (EMS; #25560) in 0.1 M PB for 2 hours on ice. After blocking, sections were labelled with the primary Anti-HA antibody (clone 3F10 Roche; ROAHAHA) at 1:350 dilution in same blocking buffer at 4°C overnight. The following day, after three washes with 0.1M PB, the sections were stained with secondary antibody, Alexa 647 fluoronanogold fab' goat anti rat (Nanoprobes; #7502) at 1:100 dilution in blocking buffer for 2 hours on ice. After labelling, nanogold particles were enhanced with silver enhancement kit (Aurion; #500.033). In short, sections were washed with Enhancement Conditioning Solution (ECS) (Aurion; #500.055) three times 5 minutes and enhanced by freshly made enhancement mixture for 60 minutes, followed by three times 7 minutes washes with ECS.
From the sections, 3mm discs of specific brain areas were punched and loaded in membrane carriers of a High Pressure Freezer (Leica ICE) together with 20% BSA in ECS and vitrified at 2050bar. The frozen samples were freeze-substituted with the quick-freeze substitution (QFS) protocol the same way as it was descripted in Baatsen et al. 2021, this time with 0.2% Uranyl Acetate (SPI; #02624-AB) in acetone. After substitution, the samples were embedded in Lowicryl HM20 (SPI; #02628-AB) inside the Leica AFS2 apparatus. 90nm sections were cut with a Leica Ultracut S ultramicrotome from the sample blocks and collected on 200mesh copper grids. The sections on the grid were stained with DAPI diluted 1:2000 in water. Followed by imaging with a 20x air lens on a Nikon C2 confocal to locate the Alexa 647 signal. After light imaging, the grids were post-stained with 4% Uranyl acetate and Reynolds' Lead and the same location was imaged on a TEM (JEM1400-LaB6, Jeol) operated at 80kV. Correlation of fluorescent signal and gold particles was done in GIMP and with the nucleoli as extra fiducial markers.

Data analysis

For plaque-induced synapse loss, 20x images were taken on Nikon TiE A1R with at least 9 images per mouse from different brain slices. From each genotype and time point at least 3 mice were used for quantification. Seven Z-stacks with 1mm thickness were obtained per image and all images were acquired using the same acquisition parameters (laser settings, 16-bit, 1024x1024 quality). The images were converted to EDF focused document in Nikon NIS-Elements Image Analysis Software. Using an automated general analysis 3 script, plaques were automatically detected and ring-like ROIs were created with increasing increments distance from the plaque (i.e. 5µm, then 10µm etc.). The number of pre-synaptic terminals in each of 5 ROIs was divided by the total area and normalized (100% being the

sum of all ROIs). After automated detection, plaques that were smaller than 100 µm² were excluded, 50-150 plaques were used for final quantification per mouse. Ha-puncta with size and intensity exclusion criteria were detected and counted. Number of puncta was divided to the area of each ROI to normalize for increased surface. The mean number of pre-synaptic puncta per ROI was calculated for all plaques per mouse. To measure the relative distribution between ROIs in different mice, the number of synapses per ROI was divided to the sum of all synapses for each mouse. The distribution of the plaque-based ROIs was plotted and shown as mean ± SEM. Dystrophic neurites were manually counted in the images used for plaque-induced synapse loss based on a largely increased size of HA-tagged synaptophysin.

For pre-synaptic density analysis, 60x (Oil objective) images were taken on Nikon TiE A1R. 35 Z-stacks were taken per image and all images were acquired with the same settings for the different time points. For synapse distribution around plaque analysis, 60x (WI) images were taken on Nikon NiE A1R. 27 Z-stacks were taken per image and all images were acquired with the same settings. Confocal microscopy multi-channel stacks were analysed through a custom ImageJ macro script implemented in Fiji (ImageJ). The complete analysis pipeline and example dataset are publicly available at:

https://github.com/vib-bic-projects/202409_Synapse_Neurite_Quantificator/tree/main

Neurites were manually annotated using ImageJ's ROI Manager based on human-specific NCAM immunofluorescence. Pre-synaptic terminals were identified through HA-positive immunostaining and segmented via StarDist algorithm, followed by size filtration and intensity thresholding to eliminate spurious detections. Pre-synaptic puncta were classified as true synapses when located within 1µm of annotated neurites. For plaque analysis, X34-positive plaques were segmented using an intensity threshold of 400 arbitrary units and a minimum size criterion of 10 µm². Multiple quantitative parameters were extracted from the segmented images, including synaptic density (number of pre-synapses per unit length of neurite) and neurite length. In plaque-containing specimens, minimum distances between plaques and both neurites and synapses were computed. All segmented images containing labelled neurites and pre-synapses were preserved for validation. Pre-synaptic clustering was assessed by calculating nearest neighbour distances between pre-synapse centroids using a custom Python script (available in the aforementioned repository). The mean synapse NND to its 3 nearest synapses is calculated for axons passing near or further away from a plaque. Bouton density was measured by calculating number of pre-synapses per unit of axon length for axons passing close to the plaque or further away.

For synapse colocalization, 24 Z-stacks were taken per image and all images were acquired with the same settings. Images were processed with Imaris software (10.2) using spot detection for HA+ pre-synapses (spot size 0.57µm) and Homer1+ post-synapses (spot size 0.47µm) with background subtraction and filter intensity mean. Nex, to determine percentage of colocalization we used a MATLAB colocalization script (Colocalize Spots XTension), with a colocalization distance threshold

of 0.5µm between the centers of the spots. To determine percentage of colocalization we divide colocalized spots by all detected HA spots * 100%. ± 200 pre-synaptic puncta were detected for each mouse.

Supplemental Figures

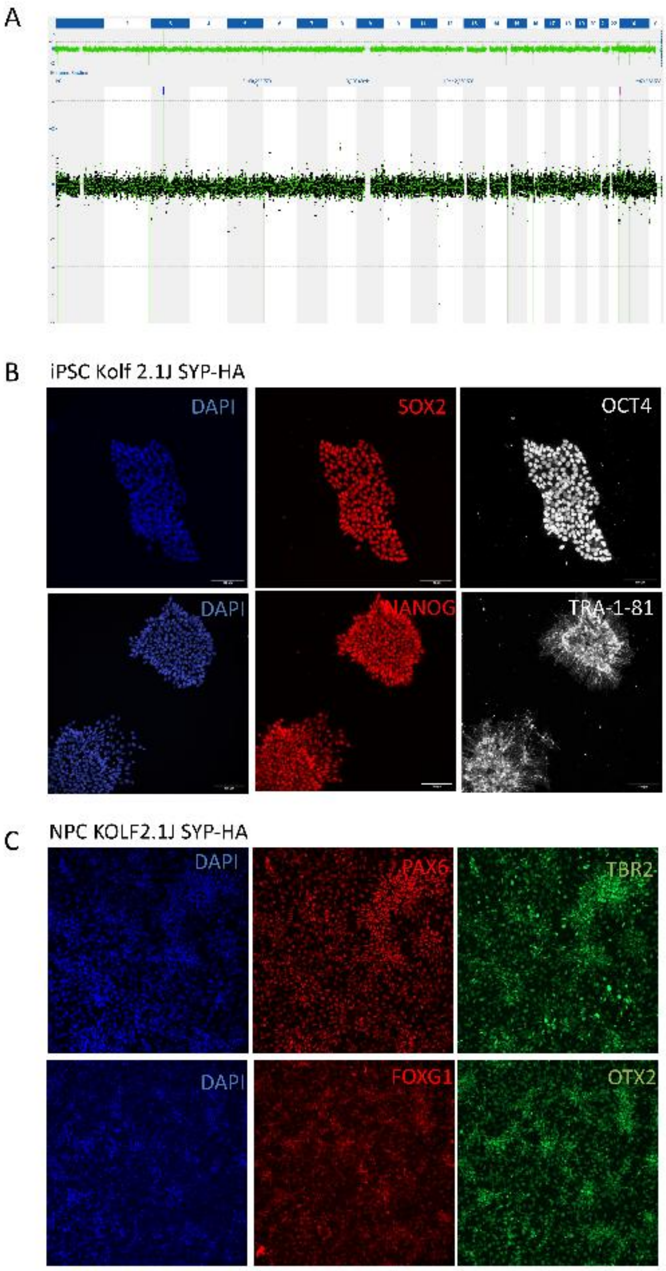

Figure S1 Quality control of correct editing of *SYP*-HA in Kolf2.1J line, related to Figure 1. (A) CGH Array shows no chromosomal aberrations induced by CRISPR/Cas9 mediated editing, (B) Pluripotency markers expressed in edited cell line, (C) and neural progenitor markers for cortical NPCs are expressed.

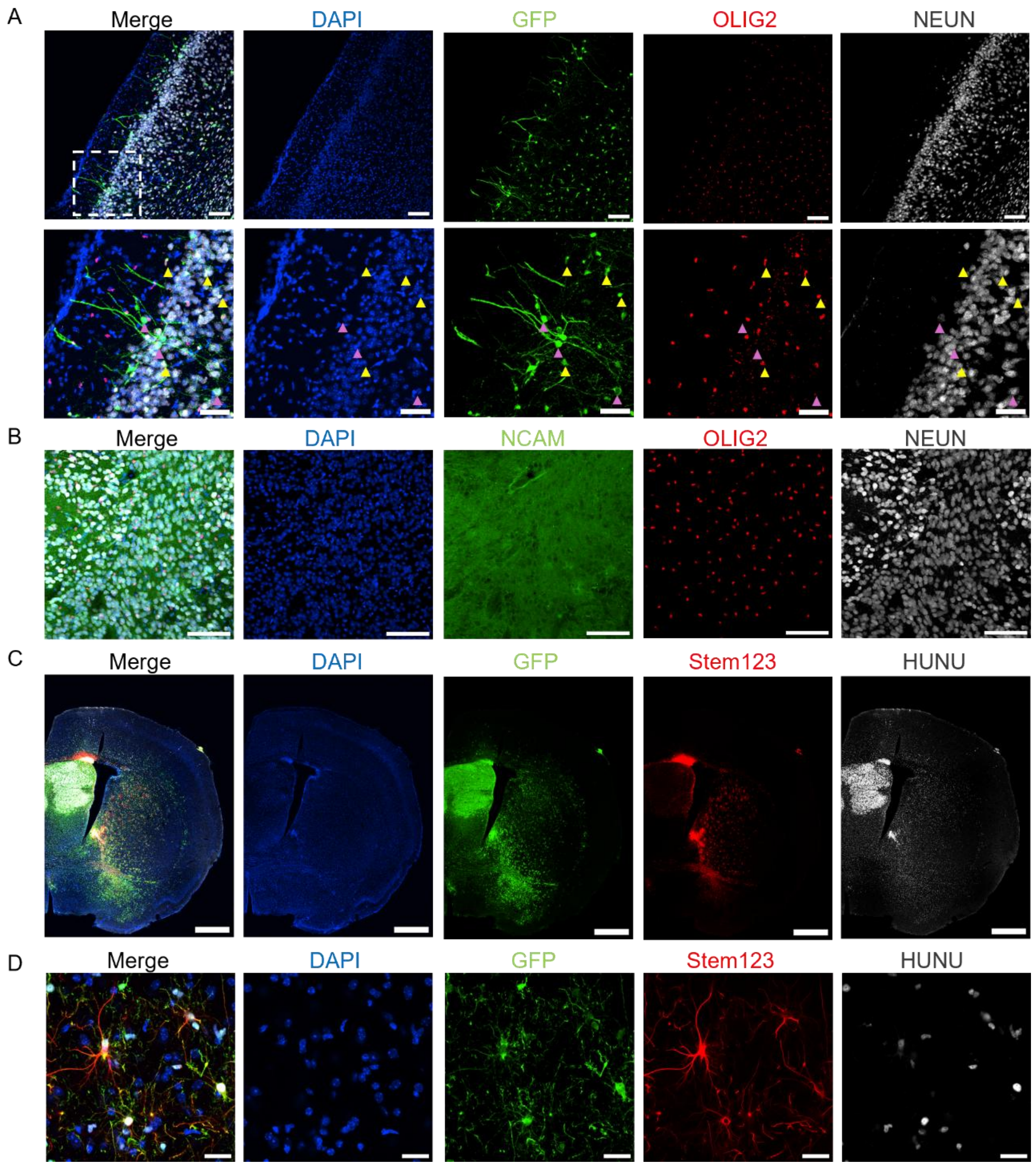

Figure S2 Graft characterization following transplantation of Kolf2.1J SYP-HA NPCs, related to Figure 2. (A) NPCs predominantly develop into human neurons (NEUN+) and oligodendrocytes (Olig+) and integrate into the brain of the host (pink arrows: human neurons, yellow arrows: human oligodendrocytes) (scale bar: 100µm and inset: 50µm). (B) The core graft region consists mainly of neurons and oligodendrocytes (scale bar: 100µm). (C) Human astrocytes can develop from the transplanted NPCs and reside in the white matter tracts and surrounding the ventricles (human astrocyte marker Stem123+) (scale bar: 1000µm). (D) Close-up of human astrocytes integrated in the host (scale bar: 25µm) (6 MPT in control and amyloid mice).

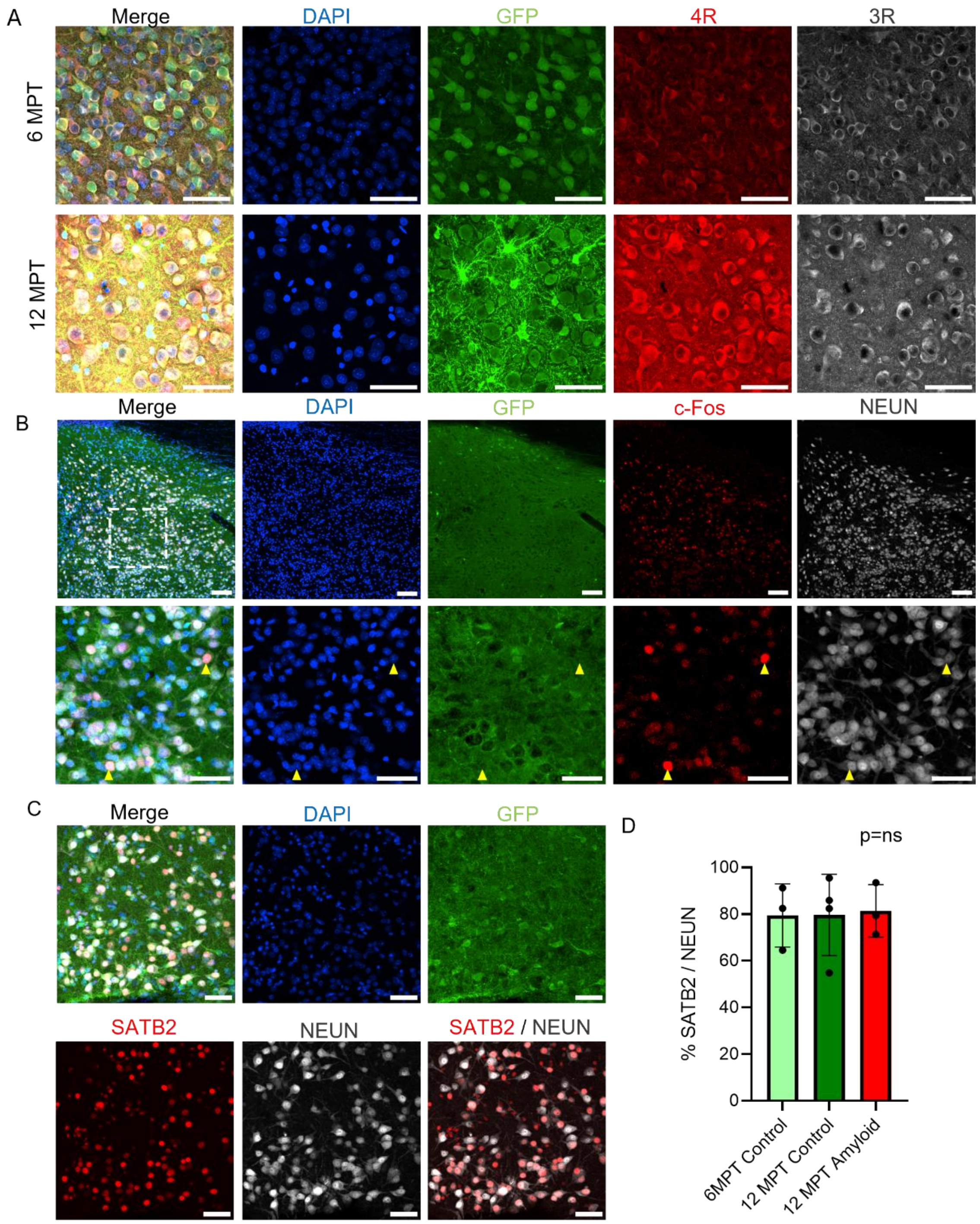

Figure S3 Human transplanted neurons develop into mature upper layer cortical neurons, related to Figure 2. (A) Human neurons express 3R and 4R tau at 6 and 12 MPT (scale bar: 50μm). (B) Neurons show c-Fos positive labelling, an indirect marker of neuronal activity at 6 MPT, yellow arrows indicate c-Fos+ neurons (scale bar: 100μm and inset: 50μm). (C) Neurons are positive for upper layer cortical marker SATB2 (scale bar: 50μm). (D) Around 80% of neurons are SATB2+ at 6 and 12 MPT and in both control and amyloid mice (n=3 mice at 6MPT in control, n=4 mice at 12 MPT in control and n=3 mice at 12 MPT in amyloid mice) one-way ANOVA F (2, 7) = 0.01639: P=0.9838 (ns p>0.5).

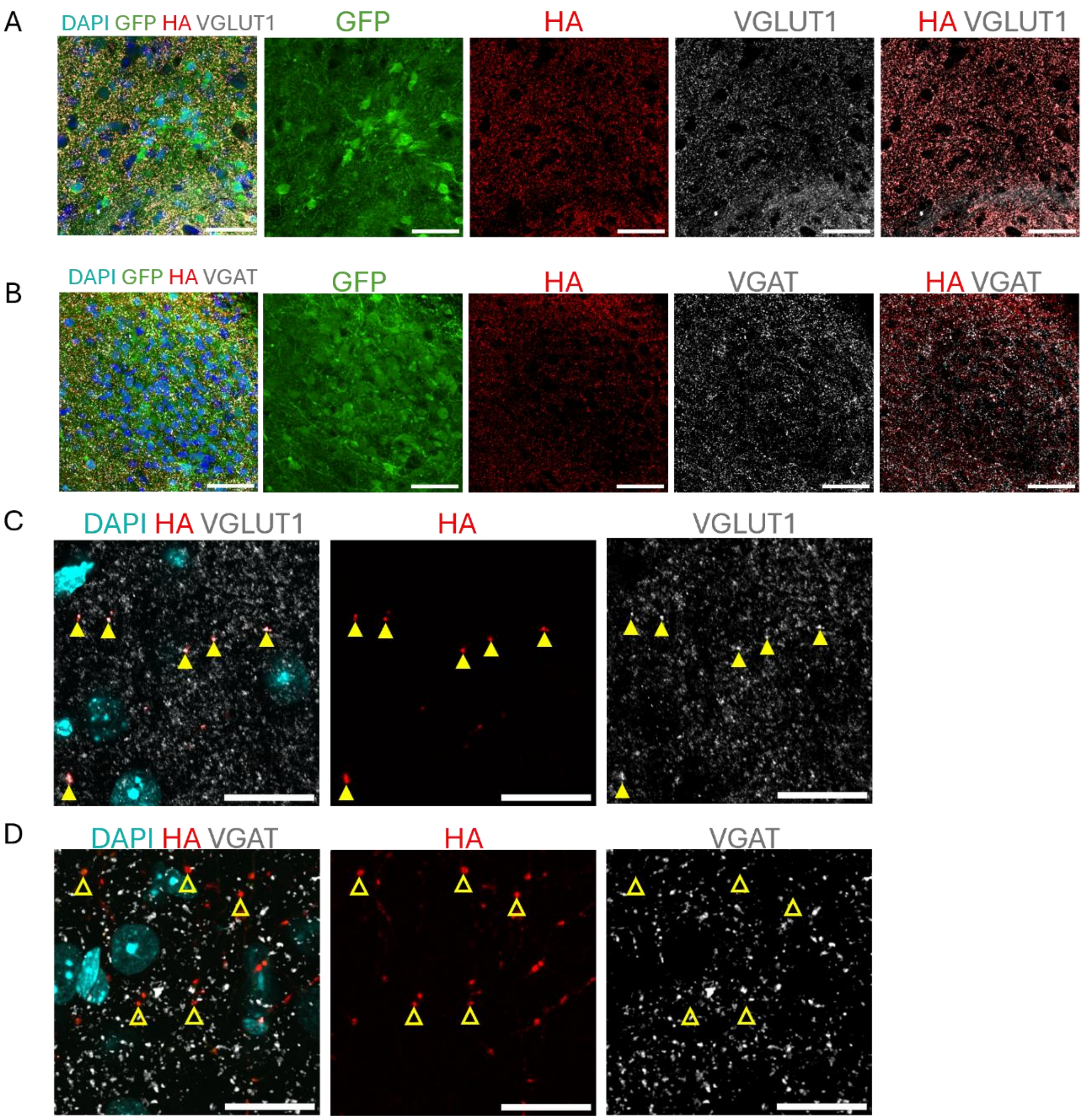

Figure S4 Human pre-synaptic puncta are predominantly excitatory (VGLUT1+) rather than inhibitory (VGAT-), related to Figure 2. (A) Dense core grafted regions of human neurons (GFP+) show abundant colocalization of human pre-synapses (HA+) with excitatory marker VGLUT1 (scale bar: 50µm). (B) Less frequently, colocalization is observed between human pre-synapses (HA+) and inhibitory marker VGAT (scale bar: 50µm). (C) Sparsely integrated human axons in the cortex show, similarly to dense core graft regions, clear colocalization of HA with VGLUT1 (yellow arrows) (scale bar: 20µm). (D) Pre-synaptic puncta colocalize infrequently with inhibitory marker VGAT (yellow arrows) (scale bar: 20µm) at 12 MPT.

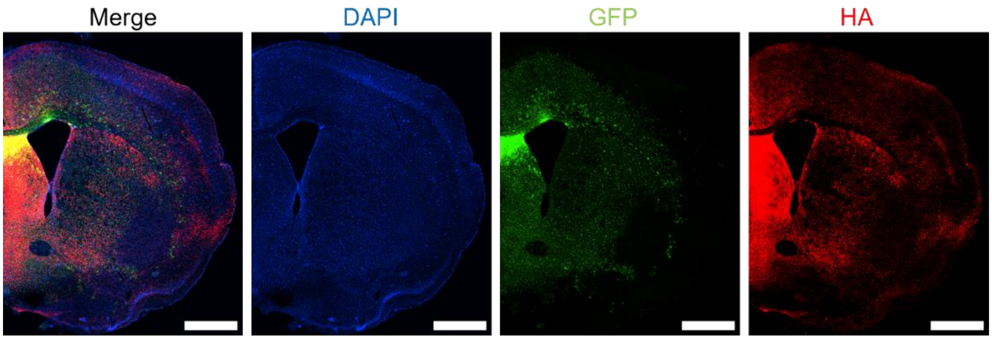

Figure S5 Overview of the human grafted region (GFP+) and pre-synaptic integration of human neurons (HA+), related to Figure 2. Example is from graft 6 MPT in control mouse (scale bar: 1000µm)

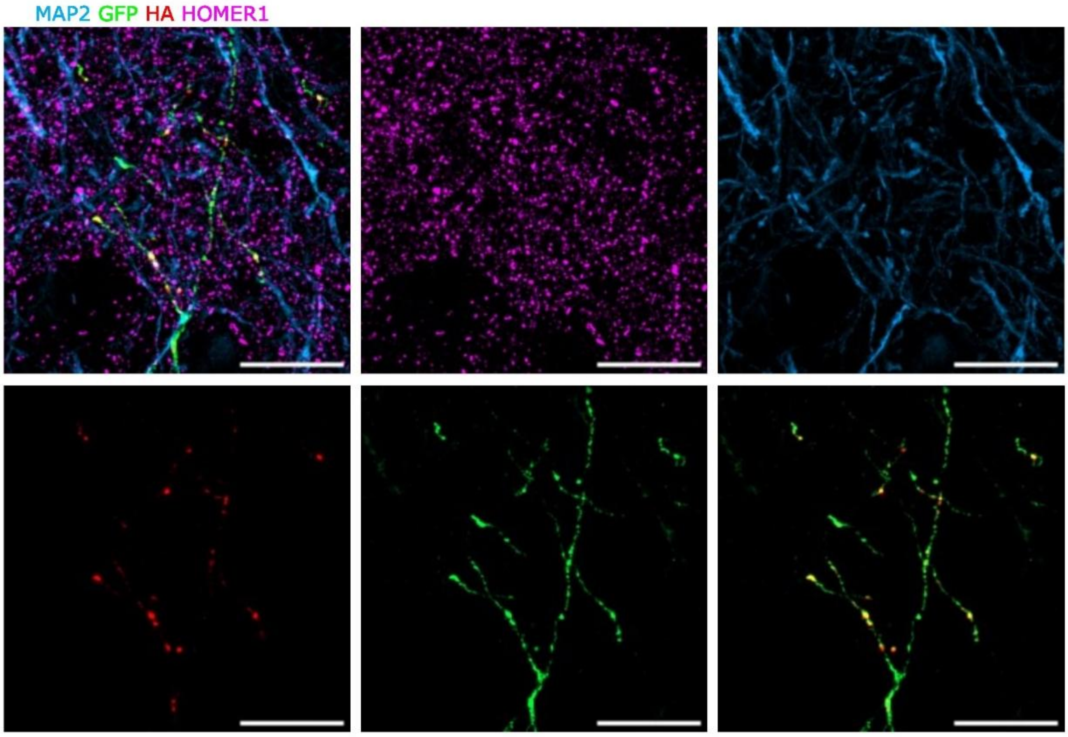

Figure S6 Human neurons form synaptic contacts with mouse neurons, related to Figure 2. Human neurons (GFP+) with pre-synaptic puncta (HA+) are contacting mouse dendrites (MAP2+ GFP-) and make synaptic contacts (HA+ Homer1+), this figure is related to Fig 2d and is an example from a control mouse at 12 MPT (scale bar: 10µm).

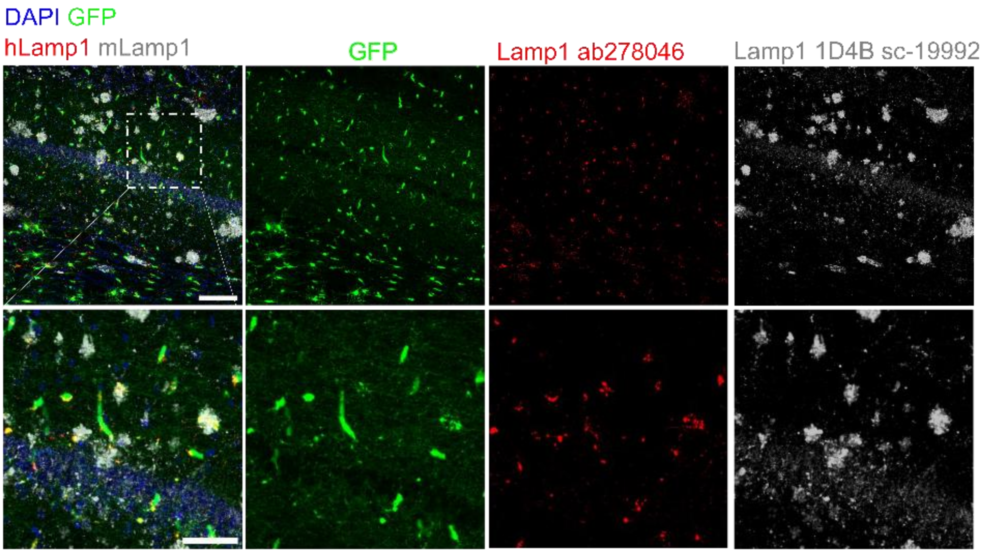

Figure S7 Human specific Lamp1 antibody, related to Figure 3. Comparison of Lamp1 antibodies led to identification of human-specific Lamp1 Ab278046 which colocalizes with human cells (GFP+) but only partly with Lamp1 (1d4B sc-19992) antibody which is more specific to mouse Lamp1.

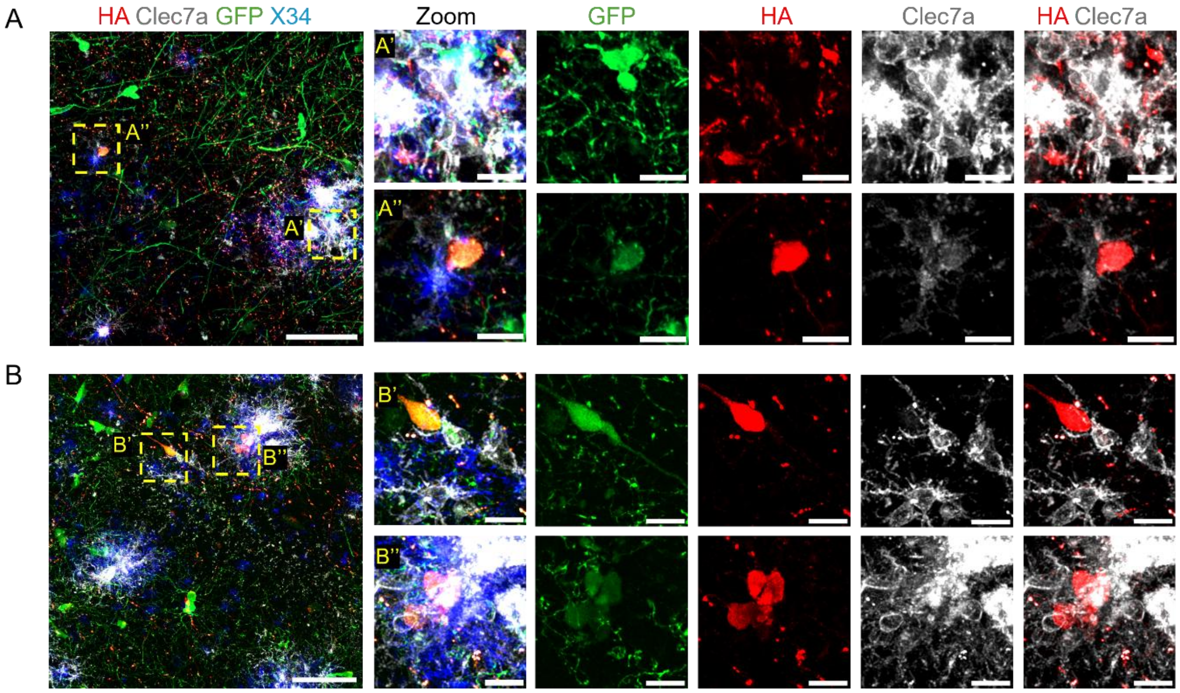

Figure S8 DAM microglia (Clec7a+) interact with human dystrophic neurites (HA+ swellings) and amyloid beta plaques (X34+), related to Figure 4. (A) Clec7a+ microglia surround both amyloid plaques (X34+) and human dystrophic neurites (HA+ swellings), note that Clec7a expression is variable ranging from very strong (A') to comparatively low levels (A''). (B) Additional examples highlight variability in Clec7a staining pattern (B' and B'') (scale bar: 50µm and insets: 10µm).

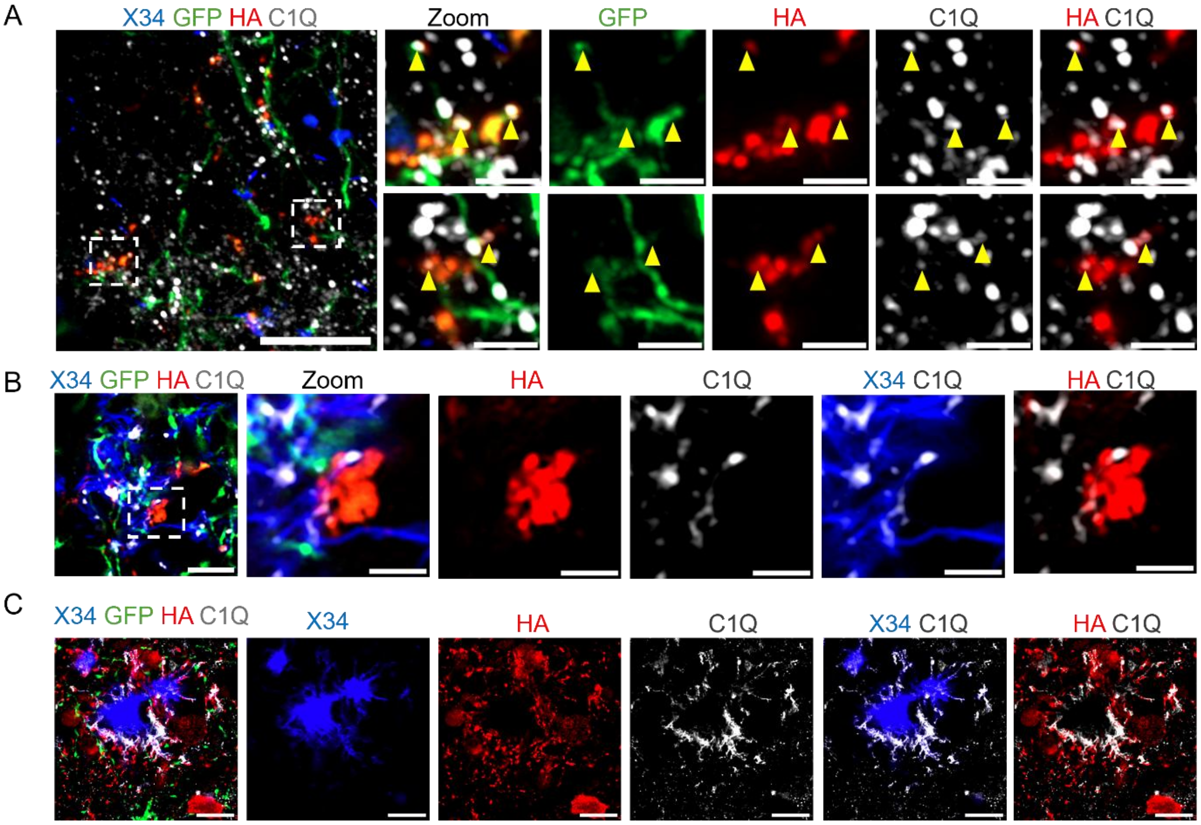

Figure S9 Human pre-synapses but not dystrophic neurites are tagged with complement component C1Q, related to Figure 4. (A) Human pre-synapses (HA+) colocalize with C1Q (scale bar: 10µm and insets: 2µm). (B) Dystrophic neurites are not detected with C1Q labelling at 12 MPT amyloid mice (scale bar: 5µm and inset: 2µm). (C) But C1Q is frequently localized to X34+ amyloid plaques (scale bar: 10µm).

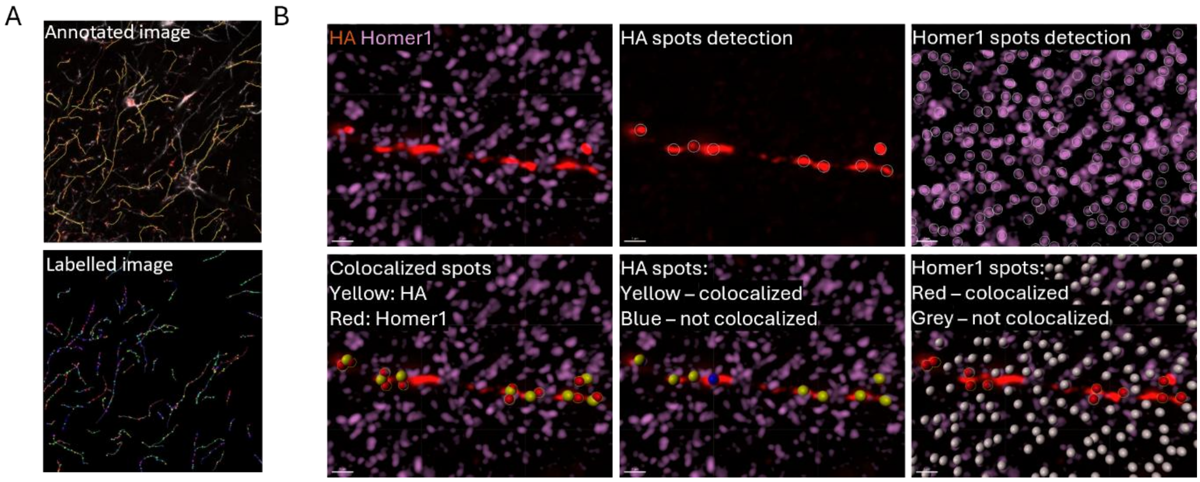

Figure S10 Semi-automated quantification of human pre-synapses and colocalization, related to Figure 5. (A) Manual annotation of human axons with automated detection of HA+-pre-synaptic boutons using in-house generated script available on GitHub (see methods). (B) Colocalization detection through Imaris (XTension). HA pre-synapses in orange and Homer1 post-synapses in purple. Following spots detection, colocalized spots are calculated based on distance threshold between the centres of the spots (HA colocalized spots are displayed in yellow and not colocalized spots in blue, Homer1 colocalized spots are in red and not colocalized spots in grey) (scale bar:1µm).
